# Communication within families about advanced pediatric cancer: A qualitative study

**DOI:** 10.1017/S1478951522001705

**Published:** 2024-10

**Authors:** Charis Stanek, Dana Garcia, Anna L. Olsavsky, Kylie N. Hill, Alexandra C. Himelhoch, Ansley E. Kenney, Lisa Humphrey, Randal Olshefski, Cynthia A. Gerhardt, Leena Nahata

**Affiliations:** 1 Center for Biobehavioral Health, The Abigail Wexner Research Institute at Nationwide Children’s Hospital, Columbus, OH, USA; 2 Department of Pediatrics, Nationwide Children’s Hospital, Columbus, OH, USA; 3 Department of Pediatric Endocrinology, The Ohio State University College of Medicine, Columbus, OH, USA

**Keywords:** Advanced cancer, Youth, Communication, Emotions, Parents

## Abstract

**Objectives.:**

This qualitative study examined how families share information and feelings about advanced pediatric cancer from the perspective of both parents and children, as well as how these perspectives vary by child developmental stage.

**Methods.:**

Participants (24 mothers, 20 fathers, 23 youth [children and adolescents]) were from a larger longitudinal study at an academic pediatric hospital. Eligible youth had advanced cancer (physician-estimated prognosis of <60%, relapse, or refractory disease), were aged 5–19 years (>8 years old to participate independently), had an English-speaking parent, and lived within 140 miles of the hospital. Interviews were completed at enrollment and asked how families share information and emotions about the child’s cancer as a family.

**Results.:**

Saturation was reached at 20 interviews for mothers, fathers, and youth. Analyses revealed 4 major themes: (A) parents managing cancer-related information based on child age/developmental stage and processing styles of family members; (B) parents withholding poor prognosis information and emotions to maintain positivity; (C) lack of personal and familial emotion sharing; and (D) emotion sharing among their family and externally. Both parents and youth endorsed themes A, C, and D, but only parents endorsed theme B. Adolescents endorsed more themes than children. Parents of children (as opposed to adolescents) endorsed theme A more.

**Significance of results.:**

Although both parents and youth with advanced cancer were generally willing to talk about treatment, emotions were not consistently shared. Perspectives varied depending on the child’s developmental stage. Clinicians should assess parent and child information and emotion-sharing needs and provide individualized support to families regarding communication about advanced cancer.

## Introduction

Despite improvements in treatment, cancer continues to be the leading cause of disease-related death in children (American Cancer Society 2020). Advanced pediatric cancer refers to disease that is recurrent, refractory, or has a poor prognosis, and can be distressing for both the child and their loved ones (Bowman et al. 2006). Honest and more frequent communication within families affected by cancer has been associated with better adjustment (Gotcher 1993). Yet, family members of a child with cancer face challenges discussing cancer with each other (Hosoda 2014). To date, most research on cancer communication has focused on adults with cancer and parent perspectives of their child’s cancer or are limited to the bereavement period, excluding the voices of children and adolescents with advanced disease (Son et al. 2019). Given communication with children and adolescents can be especially complicated in the context of advanced cancer (Feudtner et al. 2019), it is important to understand their unique communication needs and preferences. Additionally, given that parent–child communication differs by the child’s developmental stage (Pecchioni et al. 2006), more information is needed on how family communication may vary by child age within this population.

It is recommended that providers encourage open and honest communication given the long-term psychological benefits for youth with cancer and their parents (Stein et al. 2019; Wiener et al. 2015). However, research suggests that parents struggle to talk to their child about cancer, and these challenges may be heightened in the context of advanced disease (Kreicbergs et al. 2004). Parents may opt to filter information to protect their child’s well-being or be wary of discussing negative aspects of the disease or treatment for fear it may be harmful to the child’s recovery (Aldridge et al. 2017). However, filtering information in attempts to mitigate harm to youth has not been justified by the literature (Young et al. 2003). Although current literature supports parent–child communication about pediatric cancer (Webster and Skeen 2012), less is known about how often that happens in practice (Zwaanswijk et al. 2007). Research suggests that many youth with cancer want to be involved in their care and desire more information about their prognosis (Lin et al. 2020; Young et al. 2003); however, research on communication preferences of youth with advanced cancer is particularly lacking. Parents’ perspectives on information sharing remain crucial, given it is developmentally inappropriate for youth (particularly young children) to have full autonomy in medical decision-making (Miller 2018). Therefore, more research on family communication is needed to understand how parents balance their child’s information preferences with their own concerns about sharing sensitive prognosis and treatment information.

Beyond sharing diagnosis and treatment information, parents of a child with advanced cancer must manage emotions around cancer, given their role as emotional support systems for their child (Son et al. 2019). Although providers might support families in sharing their emotions, some parents have expressed a disinterest in discussing their feelings with providers (Young et al. 2013) and want to be the one to navigate emotional conversations with their child (Sahler et al. 2000). Therefore, parents may need guidance on how to initiate these conversations themselves. To date, research on emotion sharing in the context of advanced cancer is limited (Gurtovenko 2019). One study found that supportive communication before and during painful medical procedures decreased levels of distress in youth with cancer (Cline et al. 2006). However, youth with cancer do not always disclose their emotional needs (Seo et al. 2021). Additionally, most research on emotion sharing about pediatric cancer focuses on parent perspectives, whereas research on emotion-sharing preferences of youth is lacking (Son et al. 2019). For adults with cancer and youth with other chronic illnesses, emotion sharing has been associated with improved mental health outcomes (Boinon et al. 2014; Morawska et al. 2015). However, most research on the emotional needs of youth with advanced cancer focuses on palliative care and has largely ignored how youth wish to process the ongoing experience, especially near diagnosis or during active treatment (Webster and Skeen 2012).

It is particularly important to understand how families coping with advanced pediatric cancer communicate, given the potential for communication to become more difficult over time (Cowfer et al. 2021). Thus far, most research on cancer communication has focused on retrospective caregiver reports (Pai et al. 2007). In addition, little is known about the content of these conversations (Kenney et al. 2021). The perspectives of youth with advanced cancer are particularly valuable, given our limited knowledge of their psychosocial needs. Thus, the aim of this study was to qualitatively explore how mothers, fathers, and youth (i.e., children, adolescents) from each of their unique perspectives share information and feelings about advanced pediatric cancer and to explore differences by child age/developmental stage.

## Methods

### Procedure

Participants were part of a larger, institutional review board–approved, longitudinal study at a large Midwestern children’s hospital examining the experiences (e.g., goals of care, decision-making, communication) of families of youth with advanced pediatric cancer. Eligible families had a child (a) with advanced cancer (defined as relapsed or refractory disease or physician-estimated prognosis <60%), (b) aged 5–25 years, (c) with at least one English-speaking parent, and (d) living within a 140-mile radius of the hospital. Of 147 families approached, 48% (*n* = 71 families) participated in the study. Study staff recruited families via phone or in person at the hospital approximately one month after diagnosis of advanced disease.

At the enrollment study visit, family members independently completed a set of questionnaires and a semi-structured interview. An 8-question interview guide was developed by the principal investigator on the project, C.A.G., who is a trained clinical psychologist. Interviews took place at the hospital, in the family’s home, or over the phone and were conducted by research coordinators trained in qualitative interview techniques. For this study, families of children >19 years old (*n* = 5) were excluded to focus on children (5–12 years) and adolescents (13–19 years). Although parents of children aged 5–12 were included, youth had to be >8 years old to provide self-reports. The average interview lengths for mothers, fathers, children (8–12), and adolescents (13–19) were 20 (SD = 9.67), 32 (SD = 18.83), 13 (SD = 3.77), and 16 (SD = 7.85) minutes, respectively. Research staff audio-recorded and transcribed the interviews. The questions analyzed in this study were as follows: *How is information about your (your child’s) health shared or discussed in the family? How does your family share feelings about what’s happening? What are these conversations like?*

## Analysis

The coding team consisted of four researchers (L.N., C.S., D.G., and A.L.O.) trained in qualitative coding. The team used Braun and Clarke’s six-step process on thematic analysis to code interview transcriptions (Braun and Clarke 2006). First, the four coders reviewed interviews for each of the subgroups (mothers, fathers, and youth) separately, starting with mothers. Second, after the first 10 interviews with mothers were reviewed, the coding team met to generate a list of initial codes. Third, initial codes were sorted into potential themes. Fourth, the coding team used the constant comparative method to refine codes as they continued to review interviews with mothers (Glaser 1965), in batches of 10. Fifth, the final list of themes for mothers were named and defined. The mother transcripts were uploaded into NVivo, and two of the coders identified and sorted the text within these transcripts that mapped onto the final themes. This 6-step process was repeated for father and youth interviews. No new themes emerged for youth or fathers that were not already endorsed by mothers. Fathers endorsed the same themes as mothers; however, there was one theme that youth did not endorse. Twenty interviews from each subgroup were initially read. Interviews were continually reviewed until saturation was reached. Sixth, exemplar quotes were identified by two of the coders to incorporate into descriptive analysis. Themes were additionally explored by child age within two categories: children (8–12) and adolescents (13–19). Lastly, themes were explored for parents within two categories: parents of children (5–12) and parents of adolescents (13–19). NVivo was used to identify intercoder reliability (kappa = 0.91).

## Results

### Participants

In total, 106 participants completed interviews (47 mothers, 22 fathers, and 38 youth). Interviews were reviewed for mothers, fathers, and youth until reaching saturation, resulting in the final coded sample (*N* = 42 families) of 24 mothers, 20 fathers, and 23 youth. In total, the samples included 10 children, 13 adolescents, 13 mothers and 11 fathers of children, and 11 mothers and 9 fathers of adolescents. The average age for mothers, fathers, children, and adolescents was 40 (SD = 6.46), 42 (SD = 6.38), 10 (SD = 0.05), and 16 (SD = 1.5) years. The sample was largely White (86% of mothers, 81% of fathers, and 90% of youth) and of moderate socioeconomic status. The primary diagnoses of youth were leukemia (*n* = 13; 31%), lymphoma (*n* = 5; 12%), brain tumors (*n* = 7; 17%), and other solid tumors (*n* = 17; 41%). The average time since initial diagnosis and eligible diagnosis were 127 weeks and 96 days, respectively. Table 1 contains a list of demographic characteristics of individuals from all families included.

### Thematic content

**Fig. 1.**
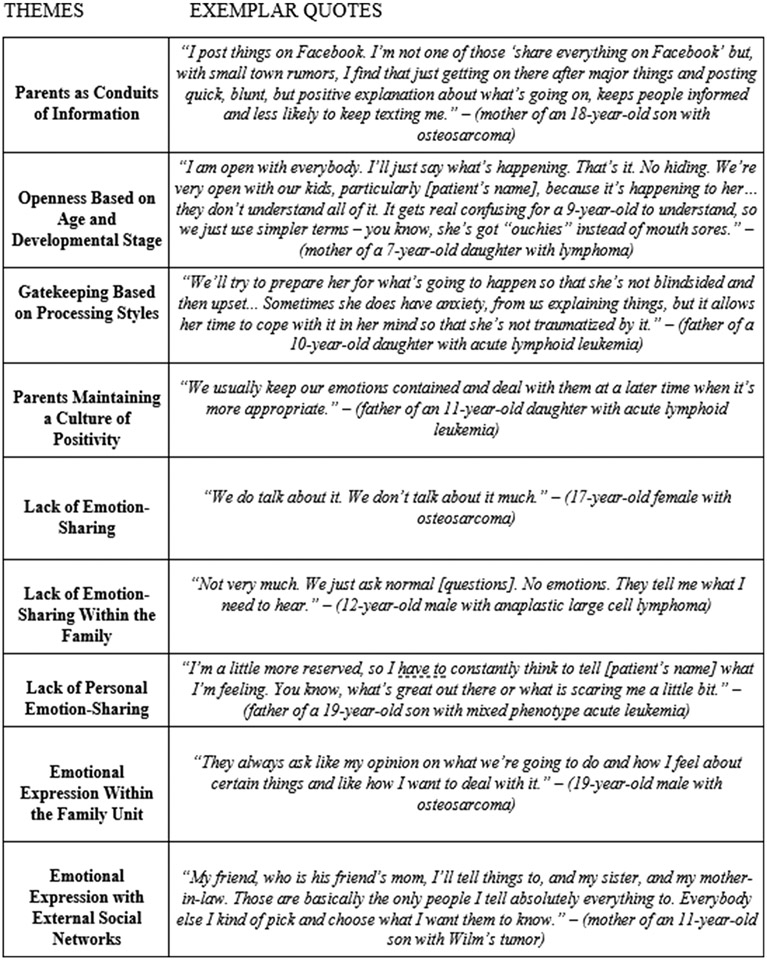
Themes and Exemplar quotes from parents and youth.

Qualitative analysis revealed four major themes: (A) parents’ role in managing information, (B) parents maintaining positivity, (C) lack of emotion sharing, and (D) emotion sharing. Parents reported all themes, while youth reported only themes A, C, and D. Theme A had three subthemes: (1) parents as conduits of information, (2) openness based on age and developmental stage, and (3) gatekeeping based on processing styles. Youth reported subthemes 1 and 2, but not 3. Theme B had no subthemes. Theme C had 2 subthemes: (1) lack of emotion sharing within the family unit and (2) lack of personal emotion sharing. Theme D had two subthemes: (1) emotion sharing within the family and (2) within external social networks. Table 2 contains frequency counts for each theme by participant. Figure 1 contains additional quotes for each theme not already reported in the results section.

### Parents’ role in managing information

Parents managed the delivery of cancer information (e.g., treatment, diagnostic information, hospital visits) and decided how to share information based on with whom they were speaking. Parents considered their child’s age and developmental stage before communicating treatment information. Relative to parents of adolescents, more parents of children spoke about adjusting their openness depending on their child’s age. Regarding sharing information with extended family and friends, parents factored in their processing style when deciding how much and what type of treatment information to divulge.

#### Parents as conduits of information

Many mothers and some fathers referenced either themselves or the other child’s parent as conduits of information from providers to their children, friends, parents, and extended family. Mothers were more often identified as the primary conduit of this information. Adolescents, but not children, also noted this theme, as one adolescent explained:

“I give permission for my mom and dad to know [treatment and prognosis information]. Mostly mom. Mom just tells dad.”

(18-year-old with sarcoma)

#### Openness based on age and developmental stage

Parents generally wanted to keep their child informed about their treatment and involve them in the decision-making process. However, many parents said they did not want to overwhelm their child with information if they were not at a developmental stage to comprehend it.

“When he was first diagnosed, he was one. He had no idea. As he’s gotten a little bit older, this is his way of life. This is all he knows, so as he gets older, we discuss more and more with him.”

(mother of a 6-year-old son with meningeal sarcoma)

#### Gatekeeping based on processing styles

Many parents edited how information is shared based on an individual’s preference for receiving information and their emotional capacity to tolerate that information. No children or adolescents commented on this subtheme. Mothers of children more often discussed gatekeeping than mothers of adolescents, whereas the child’s developmental stage had no bearing on fathers’ discussion of gatekeeping. Parents shared differences in processing information between themselves and their partner, as well as their childrens grandparents or extended family. As one mother said:

“As far as um grandparents and ones close to us, we share with them pretty quickly what’s going on. We probably keep out some of the bleak statistics, but we give them a general feel of what’s going on.”

(mother ofa 19-year-old son with osteosarcoma)

### Parents maintaining a culture of positivity

Many parents (of all youth) tried to maintain hope when discussing their child’s cancer as a family. This was acknowledged by a few adolescents but no children. Parents attempted to avoid discussing bad prognoses to protect the feelings of their children. As one mother said:

“We don’t even talk about the possibility of death. We just don’t … I don’t even tell them the odds of the chemo working or any of that. We just don’t talk about that at all. We just say that [child’s name] is going to get better, and we have to do this for that to happen.”

(mother of an 11-year-old son with Wilms tumor)

### Lack of emotion sharing

Many youth and parents mentioned a general lack of emotion sharing within their family. Parents worried that if they showed distress, that would imply a cause for concern. Some parents and youth mentioned a general, personal disinterest in emotion sharing and that they typically did not choose to share emotions with their family members. Although adolescents more often talked about lack of emotion sharing (in both contexts) than children, parents of youth in both age groups reported on this theme similarly. Some parents and youth mentioned both themes of emotion sharing and lack of emotion sharing at different points throughout their interviews.

#### Within the family unit

Youth reported that their family did not feel the need to constantly discuss feelings. Some parents prioritized discussing treatment information (e.g., appointments, medications, decision-making) over feelings. As one father said:

“There’s a lot of running and catching up and a lot of stuff… but in terms of talking about it, I think we talk, but not a lot.”

(father of a 5-year-old male with acute myeloid leukemia)

#### Lack of personal emotion sharing

Some of the references to lack of emotion sharing were specific to individual family members’ preferences for emotion sharing. Both parents and youth expressed a lack of personal emotion sharing and how that influenced conversations they had as a family. As one older child said:

“I don’t talk to anyone about it and no one talks too me.”

(14-year-old male with diffuse intrinsic pontine glioma)

### Emotion sharing

Although emotion sharing was uncommon in some families, other families discussed how they were feeling more frequently. Emotion sharing occurred for some families within the family unit, whereas for others, emotional support was more commonly found through external support systems, such as friends, external family, and larger pockets of community. Despite differences in emotion sharing among families, parents generally encouraged their children to find their way back to hopeful and optimistic thoughts.

#### Within the family

Many parents and adolescents, and some children, discussed sharing emotions within the family. When youth chose to share their feelings, it was often in the context of a specific appointment or symptom that they were experiencing, instead of a general conversation about their experience with cancer. When youth chose to talk to their parents, parents were receptive to discussing and processing the experience with their child.

“They usually express their feelings based on if I’m kind of in an emotional state. If I’m really like – not giving up per se – but in a tough situation where I’m in pain or not feeling the best, I express feelings towards it, and they tell me it’s going to be okay and that I’m going to make it through.”

(16-year-old male with osteosarcoma)

#### External social networks

Many mothers, and some fathers and youth, also sought social support outside the immediate family, such as through friends, external family members, and wider, cancer-focused support communities. Sometimes, this support was used as an alternative when emotion sharing was not common in the household. Other times, external support was a supplemental form of coping. Notably, no children or fathers of children mentioned seeking support externally. Mothers often reflected on the influence of friends and their parents on their emotional adjustment to their child’s cancer diagnoses.

“We try not to cry a whole lot, especially me in front of them. If I need time to do that, I’ll walk away or me and my mom will go talk somewhere.”

(Mother of a 9-year-old son with lymphoma)

### Discussion

Through the voices of mothers, fathers, and their children with advanced cancer, we gained a clearer picture of how families share cancer-related information and their feelings about the disease. This study adds unique insights on family communication by incorporating perspectives directly from youth and including father perspectives, which are both uncommon in pediatric cancer research. Findings indicate that parents consider multiple factors when navigating conversations pertaining to cancer, including their child’s age/developmental stage, their family members’ processing styles, and potential threats to their child’s well-being from disclosing their emotions. Many youth noted that their parents openly shared cancer-related information, while reports from both parents and youth suggested that emotion sharing was not consistent across families. Finally, there were some themes not discussed by any children, which may be more reflective of the barriers to interviewing younger children (Irwin and Johnson 2005), as opposed to actual differences based on child age/developmental stage.

Research has described variability in youth communication preferences and their involvement in decision-making (Hubbard et al. 2008; Zwaanswijk et al. 2007). Our findings align with other studies, which show that parents manage treatment information and are cautious about sharing poor prognosis information to protect the well-being of their child (Young et al. 2003), despite research indicating that youth feel isolated when they feel restrained to speak openly about their prognosis (Hilden et al. 2000). As expected, parents of children (as opposed to adolescents) were more likely to discuss how they consider their child’s age when deciding how much information to disclose about their prognosis and treatment. Mothers more often mentioned gatekeeping information based on family members’ processing styles than fathers. This is possibly because mothers were identified more often as the managers of information. Although youth were not directly prompted about their satisfaction with family information-sharing, none of them expressed concern about the amount of information relayed to them. It is possible that parents in this study were more open than the average parent or that youth were more satisfied because their parents were filtering information. Expectedly, adolescents spoke more than children about their parents being the conduits of information and being open about cancer-related information, possibly due to having a greater awareness of the role of their parents in filtering information. No adolescents or children mentioned parental gatekeeping of information, again, possibly due to a lack of awareness of this occurring. Given the diversity in responses across families, this study additionally highlights the need to seek out individual preferences of youth and their family members on information sharing as a family unit.

To date, limited research has explored how families share feelings with each other about their child’s cancer (Harris et al. 2009). Even less is known about emotion sharing in the context of advanced disease, despite the added emotional distress for youth and caregivers (Bowman et al. 2006). In alignment with previous research (Hooghe et al. 2020), many parents reported concealing their emotions due to perceptions of their emotions distracting them from their child’s needs and efforts to maintain positivity for their child. Given this concealment from parents, expectedly, few adolescents and no children highlighted this theme. Instead, fathers and mothers sometimes shared emotions with each other rather than with their child or child’s siblings. Mothers additionally mentioned leaning on external social networks to maintain optimism around their child. There were no notable differences in emotion-sharing practices for parents of children with cancer compared to parents of adolescents with cancer. While adolescents were more likely than children to discuss lack of emotion sharing, they also more often commented on occurrences of emotion sharing. It is possible that these differences reflect more detailed responses from adolescents, rather than distinct preferences between developmental groups.

The implications of emotion sharing within the context of cancer is under-researched (Wang and Wei 2020). Emotion sharing in general populations has shown to alleviate distress and contribute to meaning-making (Pennebaker et al. 2001), but that emotion-sharing benefits can be context dependent (Rimé 2009). Most youth shared few details about the frequency or quality of emotion sharing within their family but also did not express wanting more family conversations pertaining to emotion sharing. In some instances, youth shared a disinterest with personal emotion sharing, consistent with research showing that youth do not wish to constantly discuss heavy content or death (Van Schoors et al. 2020). Yet, further research is needed to examine how frequency of emotion sharing relates to psychosocial outcomes.

### Limitations

Several limitations should be considered when interpreting the findings of this study. Data are from one pediatric hospital, with a sample of people who are mostly White and have moderate incomes. To better understand the challenges of these conversations, families with diverse backgrounds must be included, especially considering cultural differences in emotional expression and regulation (Scherer and Wallbott 1994). Families who participated in this study may have been biased toward divulging more information, given their willingness to discuss cancer openly with research staff. Given the limited number of youth within each age range, and the disproportionately less themes mentioned from children, we cannot make generalizable claims about preferences based on age. Further research should explore when information sharing is developmentally appropriate and the risks and benefits of emotion sharing with pediatric cancer populations. Lastly, this study included youth after initial diagnosis or a recent relapse, which are unique experiences and therefore may have influenced family responses. Further research should longitudinally examine differences in emotion sharing and information sharing needs across different diagnoses, treatments, and times since diagnosis or relapse.

### Clinical implications

General psychosocial recommendations for youth with cancer include communicating openly about their cancer with their family (Aldridge et al. 2017); however, recommendations specific to emotion sharing as a family unit are limited. It is important for clinicians to listen to individual family needs instead of forcing emotion sharing in contexts where it is unhelpful. Talking about emotions may be particularly important for families with a child with advanced cancer, given research that finds regret among bereaved parents who avoided talking about death (Kreicbergs et al. 2004). Some research also suggests benefits of alternative types of coping instead of emotion sharing, considering that constant emotion sharing can become overwhelming for families (Wittenberg et al. 2017). Tailored counseling strategies and interventions are needed to help families determine their own information sharing and emotion sharing preferences, as well as understand the preferences of their children.

## Conclusion

This study is one of the first to examine family communication among families of youth with advanced cancer. Notably, this study adds valuable input from the youth’s perspective, which has thus far been under-reported in advanced pediatric cancer. As expected, parents take into consideration the potential risks to their child when revealing treatment information but strive to keep their child included in these conversations. Youth did not report concerns about their level of involvement in care and expressed both experiences of emotion sharing and lack of emotion sharing within their family. Psychologists can play a crucial role in supporting parents by developing interventions to help them understand their child’s information sharing preferences and emotional needs, particularly how to adjust conversations based on their child’s unique developmental stage and processing needs. Pediatric psychologists should offer to facilitate these conversations during routine checkins to alleviate pressure felt by parents in navigating these conversations alone.

## Figures and Tables

**Table 1.** Family demographic characteristics (*N* = 42)

	Mother (*n* = 35)	Father (*n* = 21)	Youth (*n* = 42)
	Mean (SD) or *n* (%)		
Age	41 (5.87)	41 (5.63)	12 (4.58)
Race			
African	1 (2%)		
Asian	2 (6%)	2 (10%)	5 (12%)
Black or African American		1 (5%)	4 (10%)
White	30 (86%)	17 (81%)	38 (90%)
Bi-/Multiracial	2 (6%)		2 (5%)
Other, European		1 (4%)	
Ethnicity			
Hispanic	0	0	0
Non-His panic	35 (100%)	21 (100%)	42 (100%)
Education			
Years completed	14.68 (2.79)	14.48 (2.93)	
Income			
Under $25,000	9 (26%)	5 (22%)	
$25,001–$50,000	6 (17%)	2 (9%)	
$50,001–$75,000	6 (17%)	6 (26%)	
$75,001–$100,000	3 (9%)	5 (22%)	
$100,001–$150,000	5 (14%)	3 (13%)	
More than $150,000	4 (11%)	1 (4%)	
Eligible diagnosis			
Initial poor prognosis			15 (36%)
Relapse/refractory disease			27 (64%)
Number of relapses (SD; range)			1.48 (0.85; 1–4)
Sex			
Male			28 (67%)
Female			14 (33%)

Note. The sample reported in this table comprises all individuals from the 44 families reported in this study to contextualize the whole family. Therefore, it includes demographic data from more individuals than only those who completed the interviews.

**Table 2.** Frequency counts of themes

Themes	Subthemes	Child (*N* = 23)				Mother (*N* = 24)				Father (*N* = 20)			
		8–12 (*n* = 10)		13–19 (*n* = 13)		5–12 (*n* = 13)		13–19 (*n* = 11)		5–12 (*n* = 11)		13–19 (*n* = 9)	
		*N*	%	*N*	%	*N*	%	*N*	%	*N*	%	*N*	%
1. Parents’ role in managing intormation	(a) Parents as conduits of information	1	10	4	31	7	54	5	45	2	18	3	33
	(b) Openness is age dependent	3	30	11	85	11	85	5	45	10	91	5	56
	(c) Gatekeeping based on processing styles	0	0	0	0	7	54	2	18	3	27	2	22
2. Parents maintaining positivity		0	0	3	23	6	46	5	45	4	36	3	33
3. Lack of emotion sharing	(a) Within the family	2	20	6	46	5	38	7	64	6	55	5	56
	(b) Personal emotion sharing	1	10	5	38	4	31	2	18	2	18	4	44
4. Emotion sharing	(a) Within the family	4	40	8	62	7	54	6	55	7	64	5	56
	(b) External social networks	0	0	3	23	5	38	4	36	0	0	3	33
